# Prediction of peptides retention behavior in reversed‐phase liquid chromatography based on their hydrophobicity

**DOI:** 10.1002/jssc.202200743

**Published:** 2022-11-14

**Authors:** Othman Al Musaimi, Oscar M. Mercado Valenzo, Daryl R. Williams

**Affiliations:** ^1^ Department of Chemical Engineering Imperial College London London UK

**Keywords:** hydrophobicity, machine learning, peptides, retention behavior

## Abstract

Hydrophobicity is an important physicochemical property of peptides and proteins. It is responsible for their conformational changes, stability, as well as various chemical intramolecular and intermolecular interactions. Enormous efforts have been invested to study the extent of hydrophobicity and how it could influence various biological processes, in addition to its crucial role in the separation and purification endeavor as well.

Here, we have reviewed various studies that were carried out to determine the hydrophobicity starting from (i) simple amino acids solubility behavior, (ii) experimental approach that was undertaken in the reversed‐phase liquid chromatography mode, and ending with (iii) some examples of more advanced computational and machine learning models.

**Article Related Abbreviations**
HFBA: heptafluorobutyric acid
LM: logarithmic model
ML: machine‐learning
QSRR: quantitative structure‐retention relationship
RPC: reversed‐phase chromatography
SVR: support vector regression

## 1 INTRODUCTION

Pharmaceutical industry has witnessed an increase in the number of approved medium‐sized molecules such as peptides [1]. Accordingly, this necessitates a proper characterization to determine the exact amino acid sequence, degree of aggregation, and stability, among others [2]. The purity of any molecule is considered a prerequisite for its structural and function studies, and subsequent applications. The preferred approach is HPLC which has been proven to be efficient in isolating peptides from by‐products, including deletion and terminated sequences [3, 4]. It is a versatile technology, with significant assay precision, and also affordable, in which most laboratories that analyze mixtures consider the HPLC as a first‐choice technique.

Preparative chromatography, a large‐scale HPLC, plays an important role in extracting/purifying biopharmaceuticals from complex mixtures. However, for efficient purification, an a priori column design process should be considered to determine the optimum operating conditions. Provided that peptide separation is considered more complicated than small molecules [5]. Information about the physicochemical nature of the separation process can be obtained from the nonlinear adsorption isotherm and is significantly affected by pH, ion pair, and organic modifier, in the case of peptides [6]. As the peptide chain becomes longer this translates to having more residues with different characteristics, fuelling various interactions such as neighbor group effects, preferred binding domain, and aggregation, among others. Several studies have helped in modeling the chromatographic process [7], optimizing the system, determining the equilibrium isotherm, and measuring the kinetic data [8].

**FIGURE 1:**
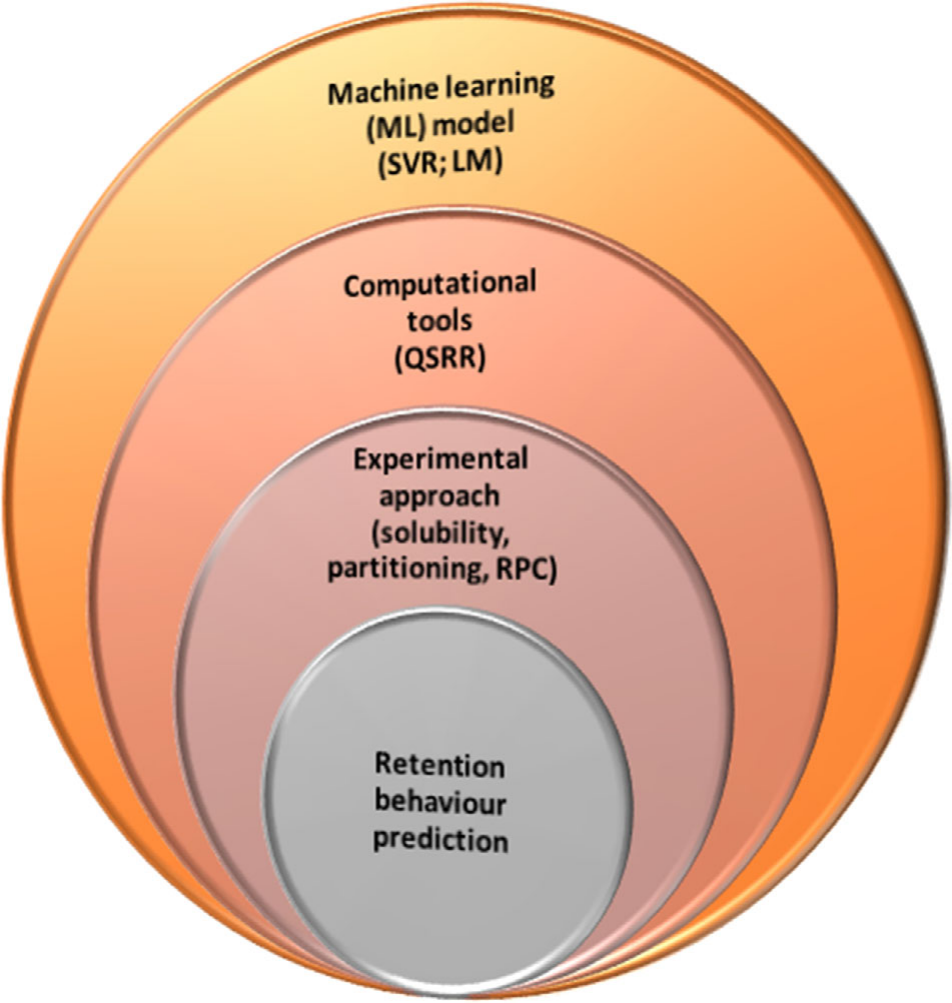
Strategies for retention behavior prediction of peptides

From a chromatography point of view, especially the reversed‐phase mode, hydrophobicity along with electrostatic, hydrogen‐bonding, and aromatic interactions, are important elements to understand how peptides interact with different chromatographic variables, such as mobile and stationary phases, solvent, temperature, etc. Hence, predicting the interaction and elution patterns saves time, effort, and cost [9]. There have been tremendous efforts invested to achieve this. Starting from simple partitioning and thermodynamic transfer of amino acids, RP chromatography (RPC), and advanced computational, and machine‐learning models (Figure 1).

### 1.1 Hydrophobicity effect

**FIGURE 2:**
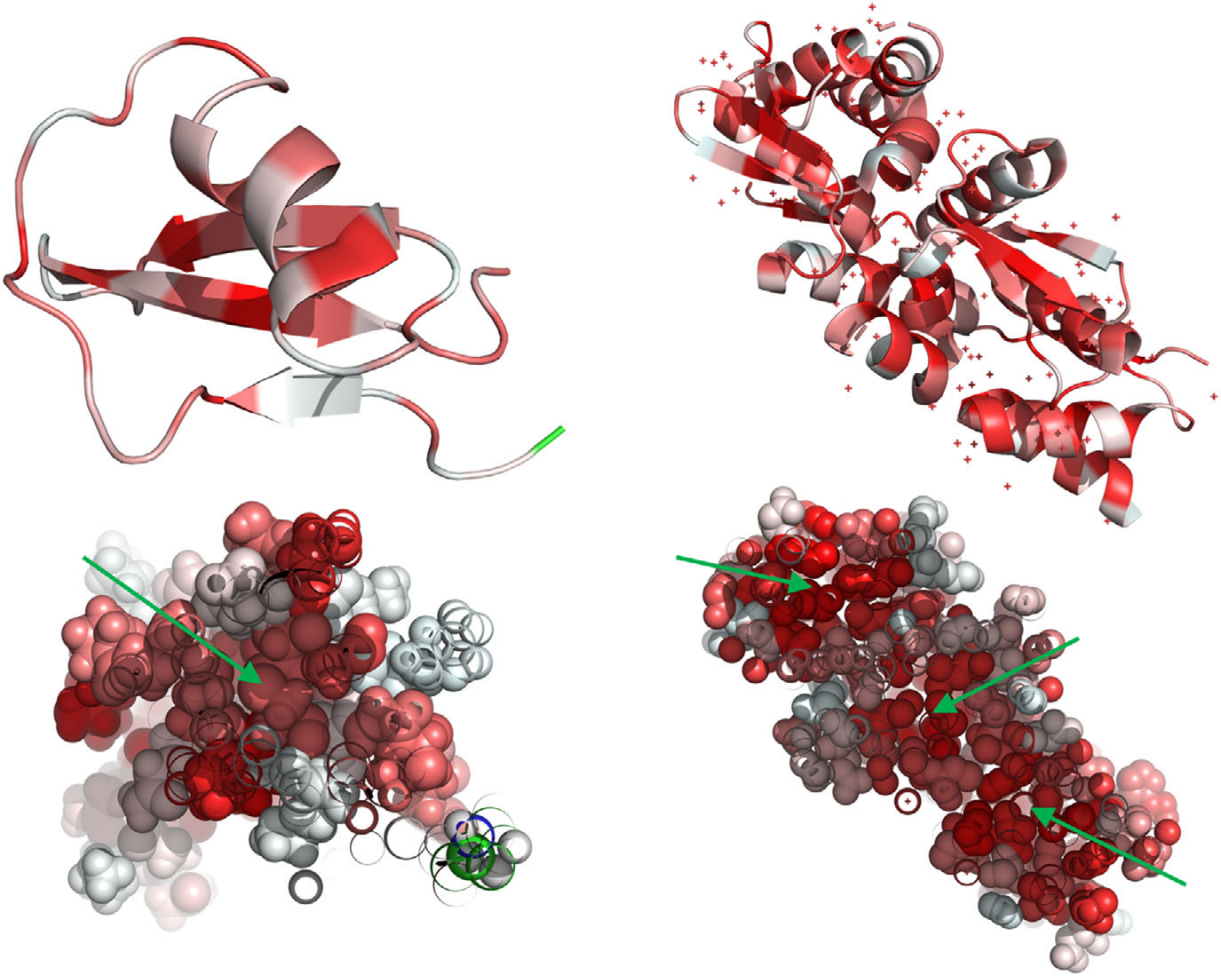
Hydrophobic cores in globular proteins in aqueous solution. Three‐dimensional structures of SWEET family transporter (left) and protein ZBTB7A (right). Red‐colored areas in both cartoons (top) and spheres (bottom) representations depict higher hydrophobicity according to the Eisenberg scale [16]. Hydrophobicity cores are pointed with green arrows.

The importance of hydrophobicity can be observed in various fields. It plays an important role in understanding protein structure and functionality [10], protein stabilization [11], peptide self‐assembly [12], hydrophobic and cationic peptide sequences are considered important for the stable non‐covalent plasmid DNA complexation and intracellular delivery [13]. It was early noted that the effect of hydrophobicity dominated the initial interactions during the folding of globular proteins over other driving forces such as steric hindrance and electrostatic interactions [14]. Thus, the protein folding process is commonly described through the hydrophobic effect, which rationalizes that in order to achieve the most thermodynamically stable state in the system, water molecules around the protein surface are displaced [15]. This enthalpic expulsion of disordered water results in the exclusion and compaction of hydrophobic residues into one or multiple hydrophobic cores within the global protein structure exposing most polar residues to the surface (Figure 2) [16]. The remaining exposed hydrophobic residues are stabilized by a cage‐like structure of water molecules, commonly called a hydration shell, which reduces the entropy of the system and keeps the protein in solution.

Kitano and co‐workers studied the effect of various amino acid solutions on the water structure by comparing the relative intensity of the O‐H stretching Raman band [17]. Although no difference was detected upon investigating amino acid solutions (Gly, Ala, Ser, Val, Leu, Ile, His, and Asn) at neutral pH, a clear effect was observed when carboxylate or ammonium ions were present. Villeneuve's group established a non‐linear hypothesis between the hydrophobicity and the antioxidant capacity of rosmarinate esters. They noticed that increasing the alkyl chain's length can enhance the antioxidant capacity (up to the octyl chain), after which, the capacity starts to diminish [18]. The hydrophilicity (the antonym to hydrophobicity) correlates very well with antigenicity, which can be predicted algorithmically [19].

In this work, we have reviewed the available studies that considered the hydrophobicity of peptides and various procedures for its determination. Having the hydrophobicity coefficients determined for amino acid residues would be utilized for predicting the behavior of the peptides in the chromatographic system and predicting their retention times.

## 2 THERMODYNAMIC TRANSFER FROM AQUEOUS TO ORGANIC

### 2.1 Amino acids solubility

The solubility of a given compound in an aqueous solution reflects the hydrophobic properties of both the compound and the containing liquid, thus hydrophobicity can be derived from solubility experiments. As protein folding occurs from the displacement of water molecules, it is possible to achieve conditions where unfolded structures are thermodynamically favorable by using a chaotropic agent that can accommodate non‐polar sidechain groups. Such agents, like urea, provide a suitable environment for the stabilization of non‐polar sidechains by forming stronger hydrogen bonds with a peptide than water does, hence, minimizing the hydrophobic effect [20, 21]. A study in ethylene glycol showed little ability to accommodate the peptide in comparison to urea [22].

Hydrophobicity scales based on amino acids solubility can be estimated based on the difference in the solvation‐free energy ($\Delta F_{t}$) when each amino acid is dissolved in a pure non‐aqueous solvent and water. Since this energy of transfer is proportional to the ratio of moles dissolved in water to those dissolved in a non‐aqueous solvent, hydrophobic species show more negative values than their hydrophilic counterparts. Although this concept provides enough information to classify amino acids according to their hydrophobic character, standardization of data is required to compare with other hydrophobicity scales. Thus, it is common practice to calculate the R group contribution ($\Delta f_{t}$) of each amino acid sidechain and the peptide backbone unit. This procedure is simply performed by subtracting the $\Delta F_{t}$ value for glycine from that of any other amino acid, as the R group for this particular amino acid, consists of only a single hydrogen [21].

It is important to mention that amino acids with poor solubility in non‐aqueous solvents (low hydrophobicity) cannot be characterized directly with this method. Instead, a regression analysis of solubility data across the entire range of water‐solvent binary mixtures should be performed to extrapolate the $\Delta f_{t}$ value at 100% non‐aqueous solvent. According to previous studies, $\Delta f_{t}$ values calculated from dilution in pure ethanol or dioxane are identical, thereby the average for these two solvents can be used to increase the accuracy of such extrapolations [23]. Table 1 shows the R group contribution to the solvation‐free energy of different amino acids.

**TABLE 1:** Hydrophobicity scale based on solvation energies from water to ethanol/dioxane. Original data retrieved from Nozaki and Tanford [23]

Amino acid	Group contribution ($-\Delta f_{t}$) cal/mol
Tryptophan (Trp) (W)	3400^a^
Nor‐leucine (Nleu)	2600^b^
Phenyl alanine (Phe) (F)	2500^a^
Tyrosine (Tyr) (Y)	2300^a^
Dihydroxyphenylalanine	1800^c^
Leucine (Leu) (L)	1800^a^
Valine (Val) (V)	1500^c^
Methionine (Met) (M)	1300^c^
Histidine (His) (H)	500^a^
Alanine (Ala) (A)	500^c^
Threonine (Thr) (T)	400^c^
Serine (Ser) (S)	−300^c^

^a^ Average values for ethanol and dioxane

^b^ Average values for ethanol, butanol, and acetone

^c^ Values for ethanol only.

According to the results obtained in this work, amino acids with aromatic sidechains showed higher hydrophobic character than the rest of the natural amino acids. The study showed that Trp is more hydrophobic than His, proving that *N*‐atom contributes to the hydrophobicity as much as the C‐atom. By comparing the hydrophobicity of Ser with Ala, and Thr with 2‐aminoisobutyric acid (Aib) (from another study), a decrease in the hydrophobicity is observed as a result of the OH group. The same effect was also observed in the case of aromatic OH (Tyr vs. Phe) however, to a lesser extent.

This study showed how the hydrophobicity could be estimated from the solubility physiochemical property, hence proving the relationship between solubility and hydrophobicity, and also it is encouraging to expand the application of this property in the chromatographic work as well.

### 2.2 Partition coefficient

In 1951, Knight utilized paper chromatography to study the movement of common amino acids and 34 peptides in phenol‐water and pyridine‐isoamyl alcohol solutions [24]. He concluded that the difference in the movement expressed as partition coefficient or retention factor ($R_{F}$), is mainly ascribed to the polarity differences of the amino acids or the peptides, giving the first hydrophobicity scale based purely on a chromatographic technique.

During the same year, Pardee established a general model to estimate $R_{f}$ values of any given peptide sequence with ±0.05 accuracy based on the individual values for amino acids proposed by Knight [25]. The mathematical equation assumes that the work required for a peptide to be transferred from one solvent to another in a binary mixture ($\Delta F_{\mathrm{peptide}}^{\circ }$) is equal to the individual work for each amino acid ($\Delta F_{\mathrm{AA}}^{\circ }$) plus the contribution of the backbone atoms (amino, carboxyl, and CONH groups) in the sequence ($\Delta F_{\mathrm{BB}}^{\circ }$). Such relationship is represented by Equation (1),

(1) $$\begin{array}{ccc} \Delta F_{\mathrm{peptide}}^{\circ } & = & \mathrm{RT}\ln{\left(\frac{1}{R_{F}}-1\right)}_{\mathrm{peptide}}\\ & = & \left(n-1\right)\Delta F_{\mathrm{BB}}^{\circ }+B+\sum_{i=1}^{n}\mathrm{RT}{\left(\frac{1}{R_{f}}-1\right)}_{\mathrm{AA}} \end{array}$$

where *n* is the number of amino acid, thus n−1 represent the number of peptide bonds, B is a correction term for the backbone contribution, and $\Delta F_{AA}^{\circ }$ is expressed as the summation of the contribution of each amino acid in the sequence.

Another common method to estimate hydrophobicity is through the partition coefficient (P) determined by the ratio of the concentration of a compound between water and a non‐polar solvent (most commonly 1‐octanol). The general Hansch model defines parameter π as the logarithmic difference between the partition coefficient between two solvents of a derivative (P_x_) and a parent molecule (P_H_) [26, 27]. This concept can be adapted for amino acid sidechains if the R group is considered a derivative of the parent backbone structure corresponding to a glycine residue (Equation (2)).

(2) $$\pi =\log P_{x}-\log P_{H}=\log P_{\mathrm{AA}}-\log P_{\mathrm{glycine}}$$

However, the poor solubility of some amino acids in the organic phase makes deriving a hydrophobicity scale purely from partitioning data a challenging task. Thus, the application of relationships between the partition coefficient and retention data from different solvent systems is necessary to circumvent the disadvantages of the Hansch method. This is possible by curve fitting p−values and $R_{F}$ transformed into *R*_M_ parameters following Bate‐Smith and Westall's relationship for unrelated solute systems (Equation (3)) [28].

(3) $$R_{M}=\log\left(\frac{1}{R_{F}}-1\right)$$

Pliška et al. considered the partitioning coefficient of reference amino acids in octanol‐water and butanol‐water and their separation in TLC using different mobile phase conditions [29]. Once the fitting parameters are obtained for both sets of partitioning data, it is possible to interpolate data to calculate π parameters from incomplete datasets. Despite the good prediction of partitioning coefficients for all natural amino acids, except for proline and cysteine, using Pliška's quadratic model, assessing hydrophobicity using the π‐scale results in an arbitrary measurement as it assumes that the interactions of other.

### 2.3 Hydrophobicity constants by Rekker

Rekker criticized the π‐scale mainly for the inaccuracy in the values of hydrophobicity for substituents [30]. Provided that the model was developed on the basis that the contribution of CH_2_ and CH_3_ to lipophilicity is the same. Thus, based on this assumption the following two analogs (C_6_H_6_ and C_6_H_5_) would have the same lipophilicity according to the π system. In addition, the π system does not count correctly for the folding phenomenon. Other groups also concluded that the terminal CH_3_ is different from the CH_2_ in the middle of the chain, hence the physiochemical properties would be also different. This implies that both groups can not have identical lipophilicity. In summary, several examples proved that fundamental errors could arise when considering the π system for lipophilicity determination.

To circumvent the previously mentioned inaccuracy in the π system, Rekker suggested considering the following formula (Equation (4)), which relies on the hydrophobicity fragmental constants [30]:

(4) $$\log P=\sum_{1}^{n}a_{n}f_{n}+c$$

*f*: the hydrophobicity fragmental constants

a: numerical factor

c: the intercept

**FIGURE 3:**
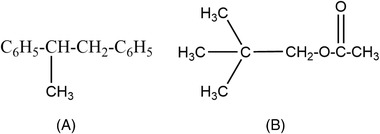
Chemical structure of two compounds to demonstrate Rekker's model.

An example of the interpretation of Rekker's work is shown in Figure 3.

In the following example, $\log P\left(A\right)=2f_{C_{6}H_{5}}+f_{CH_{3}}+f_{CH_{2}}+f_{\mathrm{CH}}$ and $\log P\left(B\right)=4f_{CH_{3}}+f_{CH_{2}}+f_{C}+f_{\mathrm{COO}},$unlike the π system, Rekker's model does count and differentiate between the CH_3_ and CH_2_.

Representative structures were chosen from literature, in which, the partitioning values in the octanol‐water were considered. After that, multiple regression analyses were carried out to establish the hydrophobicity constants (Table 2).

**TABLE 2:** Hydrophobicity constants of the amino acids by Rekker [30]

#	Name	Tri‐coded amino acid	single‐coded amino acid	Σ *f*(rel.)
1	Tryptophan	Trp	W	2.31
2	Phenyl alanine	Phe	F	2.24
3	Leucine	Leu	L	1.99
4	Isoleucine	Ile	I	1.99
5	Tyrosine	Tyr	Y	1.70
6	Valine	Val	V	1.46
7	Methionine	Met	M	1.08
8	Cysteine	Cys	C	0.93
9	Proline	Pro	P	1.01
10	Alanine	Ala	A	0.53
11	Lysine	Lys	K	0.52
12	Glycine	Gly	G	0.00
13	Aspartic acid	Asp	D	−0.02
14	Glutamic acid	Glu	E	−0.07
15	Histidine	His	H	−0.23
16	Threonine	Thr	T	−0.26
17	Serine	Ser	S	−0.56
18	Asparagine	Asn	N	−1.05
19	Glutamine	Gln	Q	−1.09
20	Arginine	Arg	R	—

The main advantages of using chromatographic techniques to determine hydrophobicity values are that no quantitative analysis is required, and the method is not restricted to a particular chromatographic system making changes in system conditions such as pH, polar strength and adsorbent material can be easily compared to other approaches.

Nevertheless, the distribution of amino acids between organic‐water system, do not express the complete retention in the reversed‐phase system, for example, the ability of the amino acids to form ion pairs with the available ions in the mobile phase (anions or cations) [6], or the interaction of the analyte with the stationary phase, etc. Thus, the HPLC technique is considered a better approach for peptide separation and specific for retention time prediction endeavors.

Various theories tried to explain the retention mechanism of protein such as salting out, hydration model, adsorption rate, and conformational changes. However, none of them received wide acceptance [31].

## 3 REVERSED‐PHASE CHROMATOGRAPHY

RPC roots can be traced back to the 50s period when Martin wants to reverse the situation of conventional chromatography to allow the separation of long‐chain fatty acids [32]. In 1984, Rivier introduced RPC to the peptide filed [33]. RPC is considered a method of choice for the separation of peptide‐based molecules due to the hydrophobic nature of the separation. Here, the non‐polar phase anchored to the resin interacts with the hydrophobic residues, separating analytes in the function of its relative hydrophobicity. These interactions can be destabilized by changing the polar strength of the aqueous mobile phase used to percolate the stationary phase by increasing the concentration of organic modifiers such as ethanol, methanol, or ACN until each analyte is eluted, affecting the retention time.

In the case of peptides and proteins, the interactions resin‐analyte can mimic to some extend the same hydrophobic interactions that drive protein folding and stability and, thus, can be used as an appropriate predictor of hydrophobic constants. Previous attempts to predict molecular properties from chromatographic data such as size, charge, and so forth have been explored. However, there is still not enough information to obtain a deterministic model. This is particularly true for hydrophobicity as the complexity of the property facilitates a change in retention by any minor variation in the RPC method or the peptide structure, causing an incorrect measurement. The main concern in the RPC is the lack of adequate retention of the polar molecules. However, considering peptide entities and their considerable degree of hydrophobicity, RPC is considered a method of choice for their separation and purification purposes [31].

## 4 PREDICTION OF PEPTIDE RETENTION TIME

The correct prediction of retention times would save labor and resources at any scale of the chromatographic endeavor. However, various intrinsic factors of the peptide of interest influence the behavior of peptides within the chromatographic system, and thus their retention time. These factors include but are not limited to; amino acid distribution along the peptide sequence, species at the *C‐* and *N‐* terminals, overall sequence length, and total charge.

Prediction of peptide retention time as well as its behavior in the chromatographic system have been one of the main goals that analytical chemists are pursuing. Various approaches and scales were developed to achieve this goal. Several experiments were carried out, and a lot of theoretical models were established. Various factors are playing crucial roles and influencing to different extents the behavior of peptides within the chromatographic system, and the way they interact with the mobile and/or the stationary phases. Including but not limited to the position of each amino acid residue, its neighboring groups, *C* and *N* terminals, chain length, and total charge, among others. Apparently, retention time prediction could save effort, chemicals, and cost and hence increase the efficiency of the whole separation process.

Here, we are presenting and discussing what has been published so far with respect to peptide retention time prediction.

### 4.1 Retention time prediction based on amino acid composition

Some research groups relied on the amino acid composition and consequently, the expected physicochemical properties, to predict the retention time.

In 1979, O'Hare and Nice investigated the potential of the HPLC by analyzing 32 hormonal peptides, in addition to 9 proteins [34]. Gradient elution was adopted in this work, utilizing phosphate buffer (0.1M NaH_2_PO_4_‐H_3_PO_4_, PH 2.1) and ACN as an organic modifier. The following columns were considered: 5 μm, 4.6 × 100 mm; Hypersil‐ODS, Nucleosil 5‐C_18_, Spherisorb ODS, LiChrosorb RP‐18, Zorbax, in addition to 5 μm, 5.0 × 250 mm C_8_.

All the investigated polypeptides were separated and resolved. The same applies to the proteins, with the exception of the three hydrophobic ones. In which, they were eluted but not well resolved. Interestingly, the authors observed that the elution (retention) pattern for the peptides (<15 residues) is correlating with constants determined by Rekker [30]. On the other hand, for longer peptides, anomalies behavior took place instead.

Various chromatographic parameters were altered to elucidate the elution mechanism and simultaneously optimize the separation process. The study suggested that isocratic elution is only suitable for a narrow range of organic modifiers, and this is also dependent on the peptide being analyzed. The polarity of the solvent used in the mobile phase proved to affect the retention time of the analytes, in addition, it could help in optimizing the process in terms of resolution and efficiency. Whereas other factors, like pH, temperature, flow rate, and type of column packing material, did not influence the separation in this study. However, as proved by different studies, these parameters do influence the separation process, for example as for the column, the silanol activation is pH‐dependent, and being activated will affect the separation process. In addition, pH plays an important role in the separation process of charged residues.

The authors suggested that hydrophobicity is the main factor that governs the separation process. Thus, they summed up the contribution from each amino acid in all peptides and proteins they investigated, using the constants determined by Rekker [30]. Thus, they were able to precisely predict the retention time for peptides of less than 15 residues. However, the prediction accuracy is being deteriorated going beyond the 16 amino acid residues. Although the procedure was able to predict some long peptides, it does not always hold true. Accordingly, a solid conclusion for such large peptides could not be drawn. This is mainly ascribed to the secondary and tertiary structures of the protein which could mask several residues from action sites and hence confer discrepancies.

The work done by Meek predicted the retention time of a peptide by summing up the contribution of each amino acid residue that assembles the peptide [35]. A study was done under various pH values of the mobile phase (2.1 and 7.4). Mobile phase A: 0.1M NaClO_4_ in water, mobile phase B: 0.1M NaClO_4_ in 60% ACN. For pH 2.1 both mobile phases contained 0.1% H_3_PO_4_, for pH 7.4 mobile phase A contained 5mM phosphate buffer (pH 7.4). Bio‐Rad ODS, 10 μm, 4.0 × 250 mm column was considered.

Meek incorporated 25 peptides in his study, including oxytocin, glucagon, Met‐enkephalin, and somatostatin, among others. A good correlation was obtained between the expected and the observed retention times (0.999 at pH 2.1 and 0.997 at pH 7.4). The outcome of this experiment revealed that the main contributor to the retention mechanism/pattern is the partitioning propensity of all amino acid residues that the peptide is made of. It is worth highlighting that all peptides included in this study did not exceed 20 amino acid residues. Hence, the generated data or model may not be valid for longer peptides. From this experiment, we can observe that in small peptides (up to 20 amino acids) there is such a correlation in the retention behavior that can be predicted based on the contribution of each amino acid. The starting retention coefficients of the hydrophilic and neutral amino acids were considered to be zero. While for the lipophilic residues, were obtained by chromatographing the oligomer of each amino acid (di‐, tri‐, tetra‐), then plotting the retention time of each peptide versus the number of the amino acid residues. In which, the slope gives the retention coefficient of each residue. The determination was carried out using different gradient programs and different columns’ suppliers to assure the reproducibility of the data. Multiple mathematical manipulations were done repetitively to assure a good correlation between the predicted and the observed retention times (Table 3).

**TABLE 3:** Retention time coefficients established by Meek at two different pHs (7.4 and 2.1) [35]

	Retention time coefficient	
Amino acid residue (*N*)*	pH 7.4	pH 2.1
Tryptophan (7)	14.9	18.1
Phenylalanine (13)	13.2	13.9
Isoleucine (4)	13.9	11.8
Leucine (9)	8.8	10.0
Tyrosine (11)	6.1	8.2
Methionine (9)	4.8	7.1
Valine (5)	2.7	3.3
Proline (10)	6.1	8.0
Threonine (5)	2.7	1.5
Arginine (7)	0.8	−4.5
Alanine (4)	0.5	−0.1
Glycine (13)	0.0	−0.5
Histidine (5)	−3.5	0.8
Cysteine (2)	−6.8	−2.2
Lysine (8)	0.1	−3.2
Serine (6)	1.2	−3.7
Asparagine (5)	0.8	−1.6
Glutamine (4)	−4.8	−2.5
Aspartic acid (5)	−8.2	−2.8
Glutamic acid (3)	−16.9	−7.5
Amino‐ (19)	2.4	−0.4
−COOH (17)	−3.0	6.9
−Amide (8)	7.8	5.0
Pyroglutamyl‐ (5)	−1.1	−2.8
Acetyl‐ (1)	5.6	3.9
Tyrosine sulfate (1)	10.9	6.5

* *N*: number of peptides used to determine the retention coefficient and contain the particular amino acid. Mobile phase A: 0.1M NaClO_4_ in water, mobile phase B: 0.1M NaClO_4_ in 60% ACN. For pH 2.1 both mobile phases contained 0.1% H_3_PO_4_, for pH 7.4 mobile phase A contained 5mM phosphate buffer (pH 7.4). Bio‐Rad ODS, 10 μm, 4.0 × 250 mm. Flow rate: 1.0 ml/min, RT.

After that, to calculate the retention coefficients, 25 peptides (included in the study) were chromatographed, and their actual retention times were recorded and compared with the expected retention time estimated from the summed retention coefficient of each amino acid and the termini (determined previously). The retention time of Met‐Enkephalin (a 5‐mer peptide) was predicted with a difference of ‐0.6 min, whereas the difference went up to 15.3 min in the case of Melittin (25 amino acids peptide). It should be noted that some other mathematical processes (addition, multiplication) were considered to compensate for various sources of errors.

The mobile phase of pH 7.4 can activate the silanol group and increase the retentivity of the peptides with a free carboxyl terminus. On the other hand, where the carboxyl terminus is masked the retention pattern will be the same with both pHs (7.4 or 2.1). Peptides with basic residues (Lys or Arg) would be eluted earlier at low pH, due to the ionization of their amine sidechain as well as the increase in the polarity of the peptide they are part of. The retention coefficients of this study showed a pronounced contribution from the aromatic and aliphatic residues on the retention process. Whereas the residues with acidic sidechains have a negative effect (decrease in the retention time of the peptide) and it is proportionate with the ionization. Basic or neutral residues have little effect on the retention process. Provided that neutral peptides were retained to a lesser extent than the charged ones.

One year after developing the retention time coefficients, Meek and Rossetti consolidated the previous findings of Meek [35]. They examined more than 100 peptides to obtain retention time coefficients out of them [36]. Again, they have achieved a correlation of (*r* = 0.98) between the predicted and the observed retention times for the new 100 peptides. In this study, retention time coefficients were investigated using perchlorate (as in the previous study [35]) in addition to phosphate (0.1M NaHPO_4_ + 0.2% H_3_PO_4_ in water) based mobile phases, mobile phase B contained 0.1% H_3_PO_4_ in ACN. The same column was considered; Bio‐Rad ODS, 10 μm, 4.0 × 250 mm, in addition to a smaller particle size one Bio‐Rad ODS, 5 μm, 4.0 × 250 mm. Despite the overall results being comparable, some differences emerged. In the previous study, an overestimation of the negative effect of Glu, Arg, and Asp was observed, in contrast to His, where an underestimation of its coefficient was recorded. A more negative effect was observed in the case of basic residues (Lys, Arg, His, and amino‐terminal). The size of the peptides has little effect on the retention time, in which, small or large peptides can elute at any retention time irrespective of their size. In our opinion, this could be ascribed to the folding pattern which could mask some regions of the peptide chain from being in direct contact with the stationary phase (Table 4).

**TABLE 4:** Retention time coefficients established by Meek and Rossetti in two mobile phases [36]

	Retention time coefficient	
Amino acid residue	NClO_4_	NaH_2_PO_4_
Tryptophan	17.1	15.1
Phenylalanine	13.4	12.6
Isoleucine	8.5	7.0
Leucine	11.0	9.6
Tyrosine	7.4	6.7
Methionine	5.4	4.0
Valine	5.9	4.6
Proline	4.4	3.1
Threonine	−1.7	−0.6
Arginine	−0.4	−2.0
Alanine	1.1	1.0
Glycine	−0.2	0.2
Histidine	−0.7	−2.2
Cysteine	7.1	4.6
Lysine	−1.9	−3.0
Serine	−3.2	−2.9
Asparagine	−4.2	−3.0
Glutamine	−2.9	−2.0
Aspartic acid	−1.6	−0.5
Glutamic acid	0.7	1.1
Amino‐	4.6	0.9
−COOH	2.2	1.6
−Amide	4.4	4.9
Pyroglutamyl‐	2.8	2.9
Acetyl‐	6.6	3.8
Tyrosine sulfate	2.4	3.7

A hundred peptides were used to determine the retention coefficient and contain the particular amino acid. In addition to the mobile phase used in the previous study by Meek [35] a phosphate (0.1M NaHPO_4_ + 0.2% H_3_PO_4_ in water) based mobile phase was used, and mobile phase B contained 0.1% H_3_PO_4_ in ACN. Bio‐Rad ODS, 10 μm, 4.0 × 250 mm, and Bio‐Rad ODS, 5 μm, 4.0 × 250 mm. Flow rate: 1.0 ml/min.

Furthermore, they studied the factors that may affect the retention time of peptides as well as the resolution among them. The behavior of peptides, where the influence of the flow rate, gradient steepness, and column efficiency (represented by the number of theoretical plates) was monitored. The effect of the flow rate was investigated using 10 peptides with various molecular weights ranging from 362 to 1000 Da. The study reported that the number of theoretical plates gets worse as either the flow rate or the molecular weight of the peptide increases. Thus, they recommend using a flow rate of up to 1.0 ml/min (max) when considering the isocratic mode. The influence of the molecular weight can be ascribed to the increased diffusion and hence broadening of the obtained peak. The authors also investigated the retention time when using either ACN, methanol, or 2‐propanol. Despite, the retention times being comparable, the peak width was worse with both methanol and 2‐propanol.

The ionic strength of the mobile phase has little effect on the retention time. Perchlorate showed to reduce the retention time of the peptides when compared with the phosphate buffer. Plus, the peak width was narrower but with negligible enhancement in the overall resolution. Decreasing gradient steepness has a pronounced impact on enhancing the resolution of complex peptide mixtures.

However, Meek was able to predict retention times for various peptides [35] a gross error was observed when it comes to the tryptic digest. For example, αT4 and αT11 eluted at the same retention time under these separation conditions. On the other hand, their predicted retention times are (‐0.1, and 27.4, respectively). This is mainly ascribed to the overestimated negative retention coefficient of the Glu residue (‐7.5 min) at pH 2.1, provided that αT4 has 3 Glu residues. As for αT11 peptide, it has 2 Asp residues with a retention coefficient of ‐2.8 min.

This problem was not noticed as the Glu and Asp residues were absent from the peptides investigated in the first study of Meek [35]. Nevertheless, in the revised data of the second work the effect of Glu and/or Asp was almost negligible, 0.7, and ‐1.6, respectively, and the correlation was higher [36]. The presence of discrepancies between the predicted and the observed retention time reaffirms that other factors do play roles in the separation process, such as conformation, size, charge, and polarity. it should be highlighted that different isomers would clearly affect the retention time, making these diastereomers separable. The addition of amino acid residue to a small peptide has a more pronounced effect than the addition of a larger one. This is attributed to the internalization of the hydrophobicity effect that results from folding and secondary structure that is often adopted by the large peptides (20 and more amino acids). In this work, the authors referred to other factors that may affect the retention coefficient like the flow rate and the gradient rate program as well.

Wilson et al. carried out a study to test the influence of various chromatographic conditions on the separation of peptides [37]. They also relied on the retention coefficients of amino acid residues that were established by Rekker (except for some residues) [30]. They estimated the retention coefficients for the amino acids using the multivariate regression analysis. The mobile phases that were considered in this study are as follows: mobile phase A: (0.125M‐pyridine/formate, pH 3.0), and mobile phase B: [1.0 M‐pyridine/acetate (pH 5.5)/60% (v/v) propan‐ 1‐ol]. All experiments in this study considered LiCrosorb RP‐8 or RP‐18 column; 5 μm or 10 μm, 4.0 × 250 mm. The best retention time prediction was for ETY tripeptide with only a 0.15 min difference, and the difference was 9.36 min in the case of a 31‐mer peptide (YGGFMTSEKSQTPLVTLFKNAIIKNAYKKGE).

The authors suggested that predicting the peptide retention time is not an easy task, and rather could be a challenging one. They have predicted the retention time for various peptides using various hydrophobicity coefficients, either from the current study or from the literature, and all did show discrepancies. Apparently, various factors could play a role other than the amino acid hydrophobicity, for instance, pH, charge, mobile phase ionic strength, and the length of the peptide chain among others. Nevertheless, the authors agreed that good information could be extracted from such studies: (i) Approximate elution time and behavior, (ii) which organic modifier could be more suitable for the elution process, (iii) assist in establishing the gradient program (Table 5).

**TABLE 5:** Retention time coefficients established by Wilson and co‐workers [37]

Amino acid residue	Retention time coefficient
Tryptophan	7.9
Phenylalanine	7.5
Isoleucine	4.3
Leucine	6.6
Tyrosine	7.1
Methionine	2.5
Valine	5.9
Proline	2.2
Threonine	−0.6
Arginine	−1.1
Alanine	−0.3
Glycine	1.2
Histidine	−1.3
Cysteine	—
Lysine	−3.6
Serine	−0.6
Asparagine	−0.2
Glutamine	−0.2
Aspartic acid	−1.4
Glutamic acid	0

Mobile phase A: (0.125M‐pyridine/formate, pH 3.0), and mobile phase B: [1.0 M‐pyridine/acetate (pH 5.5)/60% (v/v) propan‐ 1‐ol]. LiCrosorb RP‐8 or RP‐18 column; 5 μm or 10 μm, 4.0 × 250 mm. Flow rate: 0.7 ml/min, RT.

In contrast to the isocratic elution, the gradient mode proved to adequately maintain the height and width of the eluted peaks with minimal tailing during the separation process. The study also proved that varying temperatures between 25 and 55⁰C do not impact the separation of peptides. While pH values can influence the separation process of peptides, this effect diminishes as the hydrophobicity of the peptide increases. Neither the alkyl chain length nor the particle size of the stationary phase has an influence on the retention pattern. However, it should be highlighted that hydrophilic peptides which elute earlier are sometimes affected by varying the length of the alkyl length of the stationary phase (C_18_ vs. C_8_), or even the temperature.

The lengths of peptides included in this study ranged from 2 to 65 amino acids. The conclusion of this study reaffirms the dependence of the chromatographic separation process on the hydrophobicity factor of the peptide. Secondary structure can also affect the elution pattern of the peptides. Peptides composed of up to 18 amino acids have no effect on retention deviation from the expected retention time, whereas larger peptides tend to elute faster than expected based on the hydrophobicity consideration only.

Brownie and co‐workers also attempted to predict the retention time for two peptides isolated by them [38]. The retention time coefficients were determined following the same iterative regression analysis methodology, which was considered by Meek. However, the obtained results were not matching with those obtained by Meek [35, 36]. The retention coefficient determination in this study was estimated based on the ACN concentration while Meek considered the elution position (retention time) of the peptides [35]. Furthermore, TFA and heptafluorobutyric acid (HFBA) were considered in this study (0.1% and 0.13, respectively), while Meek considered NaClO_4_ as a chaotropic agent. In this study, Waters C_18_ μBondapak column was considered.

The authors tried to predict the retention times of the peptides based on Meek's model at pH 2.1. Surprisingly, a rather poor correlation was obtained either with the first model by Meek [35] or even the updated one by Meek and Rossetti [36]. Again, the reason could be ascribed to the difference in the mobile phase composition, where the perchlorate buffer was considered by Meek and Rossetti, while Brownie considered TFA and HFBA. Moreover, a distinct difference in the columns that were used by both groups. Nevertheless, the odd data obtained based on Meek's model (negative predicted retention time) gave the authors such a hint that this molecule would be eluted rapidly, which again, consolidates the importance of Meek and later Meek and Rossetti models [35, 36]. Hence, a modification to Meek's model was considered in this work.

25 peptides were investigated and after determining their retention times, attempts were carried out to determine the retention coefficients of the 20 proteinogenic amino acids and additional six other functional groups. As the authors previously proved how the perfluorinated carboxylic acids play an important role in the eluting pattern of peptides [39], in this study they have considered two different ion pair reagents of this family (TFA and HFBA). The concentration of ACN was considered to calculate the retention time coefficient and take into consideration the dead volume of the HPLC (by monitoring the disturbance of the UV baseline at 210 nm, which happens once the ACN is mixed with the aqueous solution) (Table 6).

**TABLE 6:** Retention time coefficients from two ion pairing reagents [38]

	Retention time coefficient	
Amino acid residue	0.1% TFA	0.13% HFBA
Trp	16.3	17.8
Phe	19.2	14.7
Ile	6.6	11.0
Leu	20.0	15.0
Tyr	5.9	3.8
Met	5.6	4.1
Val	3.5	2.1
Pro	5.1	5.6
Thr	0.8	1.1
Arg	−3.6	3.2
Ala	7.3	3.9
Gly	−1.2	−2.3
His	−2.1	2.0
Cys	−9.2	−14.3
Lys	−3.7	−2.5
Ser	−4.1	−3.5
Asn	−5.7	−2.8
Gln	−0.3	1.8
Asp	−2.9	−2.8
Glu	−7.1	−7.5
Amino	4.2	4.2
Carboxyl	2.4	2.4
N‐Acetyl	10.2	7.0
Amide	10.3	8.1
*O*‐Phospho	−2.4	−4.1
N‐Glyco	−8.0	−6.5

Mobile phase A: 0.1% TFA or 0.13% HFBA in water, mobile phase B: ACN. Waters C_18_ μBondapak column. Flow rate: 1.5 ml/min.

They highlighted that the set of the developed retention time coefficients is only valid on their system, column, and conditions. Thus, any group that wants to carry out such work, will have to establish its own data. Plus, the approach is applicable for small peptides (up to 50 residues). A great correlation between the predicted and the actual retention time was obtained. Acetyl δ‐Endorphin (1‐26) is a 26‐mer peptide, the authors were able to predict its retention behavior with a difference of 0.4 min when using 0.1% TFA containing mobile phase and 0.9 min with 0.13% HFBA. ACTH (1‐39) is a 39‐mer peptide where its retention behavior was efficiently predicted with a difference of ‐0.1 and 0.6 min when using 0.1% TFA and 0,13% HFBA, respectively.

Sasagawa et al. [40] monitored the behavior of 100 peptides in the RPC, using aqueous 0.1% TFA as an ion‐pairing reagent and Bondapak C_18_, 4.0 × 300 mm column. While O'Hare and Nice [34] as well as Meek [35] observed a linear relationship between the observed retention time of short peptides (<15 residues) and the summation of their amino acids constants obtained from the modified Rekker's constants (where the hydrophilic amino acids were slightly modified), Sasagawa and co‐workers observed an exponential relationship. Furthermore, considering Meek's constants in this work gave a poor correlation, too (r = 0.78).

As discrepancies are observed with both Meek and Rekker approaches, new retention time coefficients were calculated in this work using the non‐linear multiple regression analysis. Initial retention coefficients from the modified Rekker's constants were considered. The obtained values (using non‐weighted regression analysis) were different from Meek and Rekker's ones. Thus, in order to have uniform data, the weighted least squares were carried out in this study and the retention times were computed and proved to be close to those reported in the literature (Table 7).

**TABLE 7:** Retention time coefficient using weighted and non‐weighted regression [40]

	Retention time coefficient	
Amino acid residue	Weighted	Non‐weighted
Tryptophan	35.8 (12)	2.34 (12)
Phenylalanine	31.4 (86)	1.71 (86)
Isoleucine	27.4 (95)	1.38 (95)
Leucine	26.4 (129)	1.34 (129)
Tyrosine	21.0 (43)	1.23 (43)
Methionine	14.5 (64)	0.85 (64)
Valine	7.9 (33)	0.48 (33)
Proline	7.4 (89)	0.38 (89)
Threonine	7.4 (111)	0.12 (111)
Histidine	8.8 (38)	0.38 (38)
Alanine	2.4 (139)	0.13 (139)
Glutamine	3.2 (59)	0.36 (59)
Glutamic acid	2.7 (198)	0.27 (198)
Glycine	4.0 (134)	0.22 (134)
Serine	1.1 (62)	0.18 (62)
Arginine	0.0 (73)	0.26 (73)
Aspartic acid	−0.1 (165)	0.10 (165)
Asparagine	−11.3 (71)	−0.45 (71)
Lysine	−3.1 (98)	0.05 (98)
Carboxymethylcysteine	32.5 (5)	1.57 (5)
Homoserine	12.3 (13)	0.23 (13)
Aminoethylcysteine	4.3 (5)	0.31 (5)
Trimethyllysine	−38.1 (9)	−1.38 (9)
Acetyl‐	12.4 (6)	0.81 (6)
Amide‐	−13.2 (2)	−0.56 (2)

Between parenthesis represent the number of amino acids used for the calculation. Mobile phase A: 0.1% TFA in water, mobile phase B: 0.07% TFA in ACN. Bondapak C_18_, 4.0 × 300 mm column. Flow rate: 2.0 ml/min.

Peptides were chromatographed and the correlation between the predicted and the observed retention times were satisfactory (*r* = 0.984 and 0.982) using the unweighted and the weighted correlation, respectively. The maximum difference in the retention behavior prediction was 2.9 min for Lysozyme (129 amino acids).

Six peptides were investigated using different gradient programs, and the retention times were correlated with the inverse of the slope of the gradient. This study helps in estimating the retention time of a peptide under any proposed gradient program. The method showed a linear relationship which is in line with Meek values. However, an exponential relationship is also there which proves the dependence of the elution process on the composition of the amino acids, as well. The differences between this study and previous studies maybe attributed to the different ranges of the peptides incorporated. Meek investigated peptides up to 29 residues. Thus, his data fit within the linear boundaries.

Aromatic and aliphatic amino acids do have a positive contribution to the retention time, which is also comparable to Meek's findings. The correlation between this study and Meek's is (*r* = 0.816), where such a low correlation can be understood as follows: (i) the different coefficients assigned to certain amino acids in both studies, for example, neutral amino acids showed small positive contribution to the retention time (except Asp and Asn), while Meek assigned negative contribution to those residues. It should be highlighted that in the second study of Meek and Rossetti [36], the negative value of Glu was revised from ‐7.5 to 0.7 min. (ii) this study used TFA as an ion‐pairing reagent while Meek used once perchlorate and once phosphate. (iii) Meek estimated his constant from 25 peptides whereas 100 peptides were incorporated in this study, so the 25‐peptide set in Meek's study maybe quite small to estimate such constants. Nevertheless, even after Meek revised his study [36], still the correlation is poor (*r* = 0.844) in the case of the phosphate system and (*r* = 0.821) in the case of perchlorate one.

Apparently, for large peptides, the conformational structure in addition to their amino acid composition must be also taken into consideration when calculating retention coefficients. This can be included by considering the weighted regression analysis approach. At last, this study showed that a steeper gradient leads to the peptide being eluted earlier.

Sasagawa and co‐workers also determined the retention coefficient of amino acid using a polystyrene‐based column; 10 μm, 4.1 × 150 mm [41]. They incorporated 47 peptides and chromatographed them at two pHs 2 and 8. The retention coefficient was computed using the best‐fit individual. Mobile phase A was either aqueous 0.1% TFA for pH 2‐, or 5‐mM ammonium bicarbonate for pH 8, 9.6, or 11, adjusted to those pHs with ammonium hydroxide, and mobile phase B was 0.07% TFA in ACN.

The correlation between the predicted and the observed retention times was (r = 0.98). Retention time coefficients were obtained by computer‐calculated regression analysis of retention times of various peptides analyzed by HPLC. A difference of 5.1 min between the predicted and the observed retention times were obtained for a 104‐mer peptide called Cytochrome C. Of note, the values using polystyrene‐based columns are different from those obtained from the silica‐based columns. This study helps in extending the application of this theory to a wider range of columns with various stationary phase compositions.

Biomimetic chromatography is a common HPLC, however, the stationary phase has immobilized protein and phospholipid to mimic the biological environment. Such stationary phases are known as immobilized artificial membrane stationary phases. It's been exploited to assess lipophilicity, protein, and phospholipid binding. Valko et al., utilized this methodology to predict the in vivo distribution and cell penetration of various linear and cyclic peptides [42]. The study proved the usefulness of HPLC in the design endeavor of peptide therapeutics. Interested readers are encouraged to refer to the following review [43]. The immobilized artificial membrane was also utilized to characterize peptides with antimicrobial activity by Greber et al [44]. The study demonstrated the reemphasized the excellent utility of the chromatographic tool to screen peptides through their interaction with the stationary phase [44].

### 4.2 Other retention time prediction models

Retention time prediction for peptides and proteins is also considered in proteomic studies. Advanced analyses and logarithms are being utilized, in addition, MS is usually coupled with the LC to deliver confidence in the obtained data. Several differences between the common chromatographic prediction work of peptides and of proteomic one, which limit the use of the previously discussed prediction methods in the proteome analysis field. For example, the number of samples, the types of termini, and most importantly, in the common chromatographic work (of peptides) there are various chromatographic conditions (mobile phase, columns, temp, etc.), and the sample is homogenous (limited components). On the other hand, in the proteome analysis field, the chromatographic conditions are generally limited, while the efforts are mainly directed to understand the influence of the sample composition on the separation process, provided that the sample is quite complicated, where a variety of components are likely to present [45, 46, 47].

Different approaches were developed for predicting the peptide retention time in RPC. (i) index‐based model which is utilized to identify and exclude the false positive peptides obtained from database search [48], (ii) modeling‐based model along with the experimental one [49, 50], (iii) machine‐learning (ML)‐based methods [51, 52].

In index‐based methods, the effect of each amino acid in a sequence is estimated using the multilinear regression of a large set of peptides with known retention times [51]. In modeling‐based methods, the physicochemical properties of the peptide are used to predict the retention times [50]. Today, the modern machine learning approaches developed are especially important here we use a training set of peptides, or similar structural oligonucleotides are used to estimate the parameters of a predefined mathematical model based on algorithms based on artificial neural networks [45, 48], and/or support vector regression (SVR) [51, 52]. Illustrative examples of the aforementioned models are discussed below.

#### 4.2.1 Index‐based model

Gilar et al. have developed a retention prediction model that can identify and exclude the false positive peptides obtained from database search [48]. The authors compared their model to the one obtained from a decoy that contains randomized peptide sequences. The model was devised considering 20 amino acids using 1500 peptides. Acceptable results were obtained for peptides of less than 20 amino acids, and this is in line with Mant et al. [53] and Krokhin's findings [54]. On the other hand, for longer peptides, an overestimation in the predicted retention time was observed. The following equation was used to predict the retention time:

(5) $$\hat{t_{i}}=\left(1-c\ln\sum_{j=1}^{20}n_{i,j}\right)\left(b_{0}+\sum_{j=1}^{20}n_{i,j}b_{j}\right)$$

ni: contribution of amino acid.

bj: amino acid retention time coefficient.

bo: the intercept in the model.

The retention time contribution is linearised by the *ln* function using an iteratively optimized coefficient (c). Retention time coefficients are shown in (Table 8).

**TABLE 8:** Retention time coefficient at pH 2.6 and 10 [45]

Amino acid	Retention coefficient/pH 2.6	Retention coefficient/pH 10
Ala	8.94	3.16
Arg	−8.90	12.05
Asn	4.26	0.73
Asp	6.43	−8.79
Cys*	− 2.66	2.52
Gln	3.15	1.73
Glu	5.64	−8.55
Gly	2.83	1.36
His	−12.59	1.89
Ile	20.28	8.69
Leu	23.65	10.71
Lys	−10.32	10.84
Met	17.63	7.94
Phe	26.37	12.48
Pro	6.51	1.86
Ser	2.46	1.99
Thr	6.18	1.03
Trp	29.81	11.39
Tyr	14.58	6.57
Val	15.19	6.40
Free term bo	11.73	−6.43
c	0.21	0.21

RT of peptides is calculated as $\left(1-c\ln L\right)\left(b_{0}+\sum b_{j}AA_{i}\right)$; L is the peptide length.

* Cysteine alkylation. Mobile phase A: water, mobile phase B: ACN, mobile phase C: 200mM ammonium format (NH_4_FA) in water for pH 10 or 400mM NH_4_FA for pH 2.6.

A good correlation was observed between the predicted and the observed retention times (*R* ^2^ = 0.93 at pH 10). Only 8% of peptides were rejected as outliers while considering the ∓20% retention time window. The majority of those that were rejected are either weakly retained peptides or highly retained ones, where the model failed to predict their retention behavior. Provided that the weakly retained peptides were eluted with the isocratic mode and even before the actual gradient program started. In addition to difficulties to predict the behavior of long peptides, which is ascribed to their secondary structure. The model was utilized to evaluate the degree of false positive identification in a proteomic experiment. Considering the ±20% retention time window, the authors were able to identify the peptide identification error rate. In conclusion, the false positives based on the outliers appear to be greater than the ones obtained by random peptide identification via a decoy database. It should be noted that the model is not able to distinguish between the falsely identified peptides which by coincidence have the same retention time as that calculated from the model. As for the retention time prediction, the model is in line with other studies that link peptide retention with their PI. In which, the ionizable amino acids contribute more to the overall retention process, either positively or negatively depending on their protonation status. Both models (high and low pH) shied great orthogonality. Provided that each module was able to identify different outliers which were not detected by the other. In conclusion, the study proved its reliability through successful protein identification at both low and high‐pH models considered in this study.

#### 4.2.2 Modelling‐based model

Baczek and co‐workers considered three bases to predict the retention time of the peptide: (i) logarithm of the sum of the retention times of the individual amino acids (Sum_AA_); (ii) logarithm of the van der Waals volume of the peptide (VDW_Vol_); and (iii) logarithm of the calculated octanol‐water partition coefficient for the peptide (clog*P*) [49]. The first base is obtained from the HPLC data, while the other two descriptors are obtained through molecular modeling methods, the authors considered the quantitative structure‐retention relationship (QSRR) [49].

(6) $$t_{R}=b_{0}+b_{1}\log\mathrm{Su}m_{\mathrm{AA}}+b_{2}\log\mathrm{VD}W_{\mathrm{Vol}}+b_{3}\mathrm{clogP}$$

where,

*t*_R_ = Retention time

*b*_0_, *b*_1_, *b*_2_, *b*_3_: Regression coefficients.

Various columns (C_18_, Amide C_16_, CN) and temperatures (40, 60, and 80°C) were considered in this study along with the following mobile phases; A: 0.12 TFA in water and B: 0.1% TFA in ACN). 98 peptides were investigated, which cover a wide range of structural diversity as well as post‐functionality modification (acetylation).

In the study, the maximum difference in the prediction accuracy was about 1.7 min for H‐MAGAAAAG‐NH_2_. The authors concluded that the best correlation between the predicted retention times using QSRR and the observed ones experimentally was obtained in the case of the non‐polar columns, whereas the worst situation was with the polar ones (Amide C_16_). They ascribed that to the various polar intermolecular interactions between the analyte and the stationary phase which make the prediction task more difficult.

The effect of varying the gradient run time was studied by changing the run time (20, 60, and 120 min), and considering one column. The peptide retention times increased as the gradient run time increased. The following data were obtained; 0.98, 2,91, and 7.06 min considering the gradient run time of 20, 60, and 120 min, respectively. Furthermore, the correlation between the predicted and the observed retention times decreased as the gradient time increased (r = 0.964 for the 20 min gradient, r = 0.951 for the 60 min gradient, and *r* = 0.913 for the 120 min gradient). Little influence was observed among the various temperatures that were considered in this study.

In conclusion, the study demonstrated the suitability of combining data from both molecular modeling methods and experimental ones for predicting the retention times of peptides other than the trial‐and‐error approach.

Another fascinating study that utilizes QSRR was done by Greber et al [55]. The authors constructed quantitative structure–activity relationship and quantitative structure–property relationship models to predict the antibacterial activity of cationic lipopeptides. The authors studied the influence of molecular descriptors on antimicrobial activity and hemolytic properties. In conclusion, the study showed that HPLC is a valuable tool to assess the lipophilicity of short‐cationic peptides, which is an important physicochemical parameter that has a vital role in various biological processes. Furthermore, the chromatographic indices could be used to predict antibacterial activity.

#### 4.2.3 Machine‐learning‐based methods

Fornstedt and co‐workers built a machine‐learning system to predict the retention time and resolution of oligonucleotides in ion‐pair chromatography [52]. They have trained two models (ion‐pair and co‐solvent) and validated them using two pseudo‐ orthogonal gradient modes plus three gradient slopes.

The authors investigated the effect of the length and nucleobase composition and sequence. In this, they designed “in silico” one million sequences of 8‐,12‐, and 16‐mer oligonucleotide sequences. The retention times were calculated using the logarithmic model (LM) developed by Gilar [56]. After that, the authors aimed to investigate the influence of the secondary structure on the retention time prediction endeavor. The study revealed that short oligonucleotides (*n* < 5) are only marginally affected by the gradient in comparison with the longer sequences. Oligonucleotides with the same lengths but different compositions showed different retention times. Thus, this indicates different sequence‐specific contributions to the retention are taking place. Different effects were observed between the different gradient modes. For example, short oligonucleotides showed better selectivity in the co‐solvent gradient mode and the opposite is true for the IPR gradient mode.

They have compared the SVR model LM, where they noticed that the SVR gives a lower root mean squared error in all cases. Furthermore, the relative differences in the root mean squared error between both models increases with decreasing the gradient slope in both gradient modes. SVR showed higher accuracy in predicting the retention time in the IPR gradient than the co‐solvent one at all gradient slopes. These findings make sense provided that the LM model was originally developed for co‐solvent gradient mode. The trained SVR is able to identify the characteristics of different separation methods as well as help in choosing the method conditions. Thus, the authors evaluated the SVR models by calculating the retention times of 250,000 random 12 and 16‐mers. They noticed an increase in the spread of the retention time distribution with the increased length of the oligonucleotide length and a decrease in gradient slope. As the spread of the retention times was higher in the case of the ion‐pair gradient, this confirms the large role of the hydrophobicity of the base pairs in this mode. To determine the purity of the final product, an accurate retention time prediction as well as peak width are required. The authors investigated the correlation between the peak width and the retention time, where they found a strong correlation in the case of IPR gradient mode. On the other hand, a rather weak correlation was observed in the co‐solvent gradient mode. Nevertheless, in both modes, the peak width has increased with the increased retention time. In both cases, except for the steepest gradient slope, the prediction error was less than 10%, and this error does decrease with decreasing the gradient slope. The authors were able to calculate the peak width of the 250,000 random unique 12‐ and 16‐mer in addition to n – 1 impurity at each gradient slope. Of note, a higher resolution was obtained between the 12/11‐mer in comparison to that of 16‐15‐mer and regardless of the sequence composition.

In conclusion, the study showed a successful construction of an ML model that can accurately predict the retention time of phosphorothioated oligonucleotides. The resolution was also determined using the retention times from the constructed ML model and the width from the linear combination of oligonucleotide GC‐content and retention time. ML model definitely helped in selecting the optimum gradient mode and slope which will help in successful separation a priori to the experimental work. The authors envisage the expansion of the model to account for any oligonucleotide modifications provided that sufficient data is provided. Furthermore, the resolution of other impurities related to the main product could be established if trained with the required retention data. Additional data such as column chemistry, particle size, and temperature among others, can be included to increase the number of possible systems to choose from.

## 5 CONCLUDING REMARKS

Despite the enormous efforts invested in the field of peptide retention time and behavior prediction, still there is a need for continued follow‐ups and developmental work to be done. Additional considerations could be investigated and utilized to facilitate predicting the behavior of peptides and hence their retention times accurately. Nevertheless, these efforts are of utmost importance to have a flavor and an idea of how a peptide would behave and interact with various components within the chromatographic system. Having such information would definitely ease the separation task. Knowing where the peptide would be eluted even an approximate, would enhance the separation process and reduce additional costs that might be incurred from the trial‐and‐error approach. Provided that there are unlimited stationary phases with different characteristics being continuously deposited in the market.

The field has witnessed advances by including more advanced and sophisticated models to account for various variables and predict the retention time accurately. However, it is to be hoped that additional studies to be continued in the field, where incorporating more detailed characteristics of every element within the separation paradigm is recommended. For example, the hydrophobicity of the stationary phase itself, particle pore size distribution, the solubility of the target peptide in the mobile phase, or the solvent of interest.

As shown in this review, in some instances it is not enough to exploit the hydrophobicity indices outside the lab, or sometimes even the experiment itself without a proper adjustment. Several factors have been shown to affect the separation process of peptide entities. For instance, the pH of the mobile phase, the overall charge of the peptide, flow rate, gradient steepness, and the polarity of the organic modifier, among others.

Finally, our group is currently working on enhancing the overall separation process, where the idea is to incorporate any possible factor that is believed to have any influence on the separation process and utilize whatever data become available to stipulate a complete method for peptide separation, which could be used later as the basis for the purification methods, too.

## CONFLICT OF INTEREST

The authors declare that they have no conflict of interest.

## Data Availability

Data sharing is not applicable to this article as no new data were created or analyzed in this study.
